# Surgeon and Surgical Trainee Experiences After Adverse Patient Events

**DOI:** 10.1001/jamanetworkopen.2024.14329

**Published:** 2024-06-03

**Authors:** Sara P. Ginzberg, Julia A. Gasior, Jesse E. Passman, Jacob Stein, Shimrit Keddem, Jacqueline M. Soegaard Ballester, Caitlin B. Finn, Jennifer S. Myers, Rachel R. Kelz, Judy A. Shea, Heather Wachtel

**Affiliations:** 1 Department of Surgery, University of Pennsylvania Health System, Philadelphia; 2 Center for Healthcare Improvement and Patient Safety, University of Pennsylvania, Philadelphia; 3 Leonard Davis Institute for Health Economics, University of Pennsylvania, Philadelphia; 4 Perelman School of Medicine, University of Pennsylvania, Philadelphia; 5 Sackler School of Medicine New York State/American Program, Tel Aviv University, Tel Aviv, Israel; 6 Department of Family Medicine and Community Health, University of Pennsylvania Health System, Philadelphia; 7 Department of Surgery, Weill Cornell Medicine, Philadelphia, Pennsylvania; 8 Department of Medicine, University of Pennsylvania Health System, Philadelphia

## Abstract

**Importance:**

Adverse patient events are inevitable in surgical practice.

**Objectives:**

To characterize the impact of adverse patient events on surgeons and trainees, identify coping mechanisms, and assess whether current forms of support are sufficient.

**Design, Setting, and Participants:**

In this mixed-methods study, a validated survey instrument was adapted and distributed to surgical trainees from 7 programs, and qualitative interviews were conducted with faculty from 4 surgical departments in an urban academic health system.

**Main Outcomes and Measures:**

The personal impact of adverse patient events, current coping mechanisms, and desired forms of support.

**Results:**

Of 216 invited trainees, 93 (43.1%) completed the survey (49 [52.7%] male; 60 [64.5%] in third postgraduate year or higher; 23 [24.7%] Asian or Pacific Islander, 6 [6.5%] Black, 51 [54.8%] White, and 8 [8.6%] other race; 13 [14.0%] Hispanic or Latinx ethnicity). Twenty-three of 29 (79.3%) invited faculty completed interviews (13 [56.5%] male; median [IQR] years in practice, 11.0 [7.5-20.0]). Of the trainees, 77 (82.8%) endorsed involvement in at least 1 recent adverse event. Most reported embarrassment (67 of 79 trainees [84.8%]), rumination (64 of 78 trainees [82.1%]), and fear of attempting future procedures (51 of 78 trainees [65.4%]); 28 of 78 trainees (35.9%) had considered quitting. Female trainees and trainees who identified as having a race and/or ethnicity other than non-Hispanic White consistently reported more negative consequences compared with male and White trainees. The most desired form of support was the opportunity to discuss the incident with an attending physician (76 of 78 respondents [97.4%]). Similarly, faculty described feelings of guilt and shame, loss of confidence, and distraction after adverse events. Most described the utility of confiding in peers and senior colleagues, although some expressed unwillingness to reach out. Several suggested designating a departmental point person for event debriefing.

**Conclusions and Relevance:**

In this mixed-methods study of the personal impact of adverse events on surgeons and trainees, these events were nearly universally experienced and caused significant distress. Providing formal support mechanisms for both surgical trainees and faculty may decrease stigma and restore confidence, particularly for underrepresented groups.

## Key Points

**Question:**

How are surgeons and surgical trainees affected by adverse patient events, and what coping mechanisms are used?

**Findings:**

This mixed-methods study surveyed 93 surgical trainees and interviewed 23 faculty and found that surgeons and trainees frequently grapple with the personal impact of adverse events. Trainees who identified as female and/or from a minority racial or ethnic background more frequently reported negative psychological consequences, and surgeons described opportunities to reduce stigma and improve support mechanisms.

**Meaning:**

These findings suggest that the negative personal impact of adverse events is underrecognized but nearly universal in surgery; more formal structures to restore surgeon and trainee well-being are warranted and may be particularly helpful for underrepresented groups.

## Teaser

This mixed-methods study examines how trainees and faculty cope with the impact of adverse patient events.

## Introduction

Medical errors are estimated to be the third most common cause of death in the US, with more than 250 000 deaths attributable to medical errors annually.^1,2^ Nearly all physicians report having made at least 1 error during their career.^3^ Despite the frequency of medical errors, it can be very disturbing for physicians to realize that they have unintentionally harmed a patient.^4^

In 2000, Wu^5^ described the experience of health care professionals who made a mistake that negatively affected a patient. Previous studies suggest that, in the wake of adverse patient events, most health care workers face negative psychological consequences, such as shame, anxiety, and second-guessing their clinical competence.^3,6,7,8,9^ Moreover, this negative personal reaction may trigger a vicious cycle of depression, burnout, and future errors.^10,11^ Ultimately, clinicians who have been involved in errors may avoid certain types of patients and cases and be more likely to leave their practices or leave direct patient care altogether.^3,4,12,13^

Given the invasive nature of surgical work, the perfectionist culture in surgery, and the frequency of malpractice claims relative to other specialties, surgeons may have unique reactions and needs in the aftermath of adverse events.^14,15^ However, little research has focused on the surgeon and surgical trainee experience or investigated how their reactions to errors may vary according surgeons’ personal characteristics or stage of career.^16,17,18,19^ Therefore, in this mixed-methods study, we sought to (1) better characterize the perceived impact of adverse patient events on surgeons and surgical trainees, with attention to potential differences related to surgeon demographics; (2) describe current coping mechanisms; and (3) identify potential opportunities for institutions to help restore surgeon well-being and promote optimal functioning in the wake of such events.

## Methods

This study was designed to capture the experiences of both surgical trainees and faculty with adverse patient events. Given pragmatic concerns about surgical trainees’ work schedules, a survey was used, whereas qualitative interviews were used to assess faculty experiences to elicit maximal depth and nuance in this sensitive subject matter. This study followed the Consensus-Based Checklist for Reporting of Survey Studies (CROSS)^20^ and Consolidated Criteria for Reporting Qualitative Research (COREQ)^21^ guidelines and was deemed exempt by the institutional review board of the University of Pennsylvania because it involved survey and interview procedures and the data were maintained and reported in a deidentified fashion.

### Definition

We defined adverse patient events as both overt errors and unanticipated complications. The rationale for this broad definition is that the distinction between these 2 types of events is often blurred, and the psychological effects on the involved clinician are often similar.

### Part A: Trainee Survey

A cross-sectional survey was administered to surgical trainees at a single academic institution. The Second Victim Experience and Support Tool, a validated instrument used in multiple health care settings, was adapted using a modified Delphi method.^22,23,24,25^ The instrument was reviewed by an expert panel via email correspondence. Six questions irrelevant to the surgical trainee population were removed, and 13 new questions were developed by majority agreement. Questions regarding the forms of support used in the aftermath of adverse events, such as free counseling sessions offered through the Employee Assistance Program, were added to inform potential future interventions. Demographic questions, including self-reported gender identity, race, and ethnicity, were added so that the study team could assess for associations between these characteristics and the personal impact of adverse patient events. The adapted survey instrument included 37 questions (eAppendix 1 in Supplement 1).

Trainees enrolled in 7 surgical programs offered by our health system (cardiothoracic surgery, general surgery, orthopedic surgery, otorhinolaryngology, plastic surgery, urology, and vascular surgery) were invited to complete the survey using a secure, web-based tool during educational conferences between September and November 2022.^26^ The first page of the survey was composed of information about the study and institutional review board approval, including a statement that completing the survey would constitute consent to participate in the study. One follow-up reminder was sent via email. Multiple participation was not captured to prioritize anonymity.

Survey data were reported using descriptive statistics. Responses that used a 5-point rating scale were dichotomized as no (“not at all”) vs yes (“a little,” “somewhat,” “quite a bit,” or “a lot”) to perform comparisons between subgroups using χ^2^ tests and Cohen d statistics.^27^ Incomplete surveys were excluded. Incomplete surveys were excluded; however, if a participant responded to at least 1 part of a multipart question, their survey was retained for analysis.

### Part B: Faculty Interviews

One-on-one semistructured interviews were conducted with surgical faculty from 4 departments at the same institution. Surgeons were purposively recruited via email, ensuring participation from both genders, a breadth of subspecialties, and multiple levels of experience. The interview guide was developed by consensus among members of the study team (S.G., J.S., and H.W.) to expand on themes introduced in the survey instrument (eAppendix 2 in Supplement 1). All interviews were conducted between June and September 2022 by a female general surgery resident who had completed coursework in qualitative research methods, had previously worked clinically or professionally with 12 of the interviewees, and had disclosed a personal interest in the topic (S.G.). All interviews took place either in the faculty member’s office or by video conference and lasted 20 to 60 minutes. Verbal informed consent was obtained before commencing each interview. The interviews were audio recorded, transcribed verbatim, and deidentified before analysis. Transcripts were reviewed and corrected (S.G.) but not shared with participants.

An applied thematic analysis approach was used.^28^ On the basis of review of the literature as well as preliminary assessment of the transcripts, an initial codebook was developed by consensus of the study team. Eight transcripts were coded independently (S.G., J.G., J.P., and J.S.), and then coders met to adjudicate disagreements (minimum Cohen κ coefficient of 0.8). Subsequently, 15 transcripts were coded by consensus between pairs of coders. The codebook was iteratively refined as additional transcripts were assessed. Codes were organized into themes within each of the study domains by coder consensus. No additional interviews were scheduled after the coders agreed that thematic saturation had been reached.

### Statistical Analysis

Statistical analyses were performed using Stata, version 17.0 (StataCorp LLC), and qualitative analysis was conducted using NVivo, version 20.7.1 (QSR International). We used χ^2^ tests with effect sizes to compare survey responses between subgroups of trainees. A 2-sided *P* < .05 was considered statistically significant.

## Results

### Part A: Trainee Survey

Of 216 eligible trainees, 93 (43.1%) completed surveys. The greatest proportion of participants came from the general surgery program (37 trainees [39.8%]), followed by otorhinolaryngology (14 trainees [15.1%]) and plastic surgery (12 trainees [12.9%]). Most respondents were in their third postgraduate year or higher (60 trainees [64.5%]). Forty-nine trainees (52.7%) identified as male and 41 (44.1%) as female; 3 (3.2%) did not report gender. Participants reported their race as Asian or Pacific Islander (23 trainees [24.7%]), Black (6 trainees [6.5%]), White (51 trainees [54.8%]), or other (any race other than Asian or Pacific Islander, Black, or White) (8 trainees [8.6%]), and 13 trainees (14.0%) identified as having a Hispanic or Latinx ethnicity. Most reported being married (37 trainees [39.8%]) or in a committed relationship (29 trainees [31.2%]). These demographic characteristics of respondents approximated the national surgical trainee population and are summarized in Table 1.^29^

**Table 1.** Demographic Characteristics of Respondents to the Trainee Survey

Characteristic	No. (%) of respondents (N = 93)
Specialty	
Cardiac surgery	6 (6.5)
General surgery	37 (39.8)
Orthopedic surgery	8 (8.6)
Otorhinolaryngology	14 (15.1)
Plastic surgery	12 (12.9)
Urology	10 (10.8)
Vascular surgery	6 (6.5)
Role	
PGY-1 resident	18 (19.4)
PGY-2 resident	15 (16.1)
PGY-3 or PGY-4 resident	29 (31.2)
PGY-5 or later resident	24 (25.8)
Fellow	7 (7.5)
Gender	
Female	41 (44.1)
Male	49 (52.7)
Prefer not to answer	3 (3.2)
Race	
Asian or Pacific Islander	23 (24.7)
Black or African American	6 (6.5)
White	51 (54.8)
Other^a^	8 (8.6)
Prefer not to answer	5 (5.4)
Ethnicity	
Hispanic or Latinx	13 (14.0)
Not Hispanic or Latinx	72 (77.4)
Prefer not to answer	8 (8.6)
Relationship status	
Married	37 (39.8)
In a committed relationship	29 (31.2)
Single	22 (23.7)
Prefer not to answer	5 (5.4)

Abbreviation: PGY, postgraduate year.

^a^ Any race other than Asian or Pacific Islander, Black, or White.

Most participants (77 [82.8%]) reported they had been involved in at least 1 adverse patient event in the past year, and 21 participants (22.6%) were involved in 5 or more events. Most trainees experienced negative emotional and cognitive consequences after involvement in adverse events. For example, 67 of 79 trainees (84.8%) endorsed feeling embarrassed, and 64 of 78 trainees (82.1%) reported rumination. Although a smaller proportion of trainees reported physical symptoms, such as trouble sleeping (20 of 78 trainees [25.6%]), many endorsed feelings of self-doubt; 65 of 78 trainees (83.3%) experienced feelings of inadequacy regarding their patient care abilities, 51 of 78 trainees (65.4%) reported feeling afraid to attempt future procedures, and 28 of 78 trainees (35.9%) had thought about quitting training. The proportions of respondents who answered yes to each question are given in Table 2.

**Table 2.** Surgical Trainees Who Responded “Yes” to Questions Regarding the Personal Impact of Being Involved in Adverse Patient Events and Representative Faculty Interview Quotations From Domain 1 (Personal Impacts of Adverse Patient Events)

Statement	“Yes” responses^a^							Representative faculty quotations
	No. of trainees (%)			*P* value	No. of trainees (%)		*P* value	
	All (N = 79)	Female (n = 34)	Male (n = 42)		Minority race or ethnicity (n = 36)	Non-Hispanic White (n = 38)		
I have experienced embarrassment from these instances.	67 (84.8)	30 (88.2)	36 (85.7)	.75	33 (91.7)	31 (81.6)	.21	“That certainly haunted me for a very long time, especially [since] I was new to the institution. I was embarrassed. There was many levels of guilt and I kind of thought that people will lose confidence in my abilities.”
My involvement in these types of instances has made me fearful of future occurrences.	70 (88.6)	33 (97.1)	36 (85.7)	.09	34 (94.4)	33 (86.8)	.26	“Every time you operate on somebody, you have the ability to hurt somebody, and how you deal with that will impact your career for as long as you’re operating.”
My experiences have made me feel miserable.	45 (57.7)	20 (58.8)	24 (57.1)	.88	25 (69.4)	19 (50.0)	.09	“I was just utterly devastated because this was my patient on my watch. And it was like one of the worst experiences of my life, having to go out and basically say, this was something that didn’t have to happen.”
I feel deep remorse for my past involvements in these types of events.	54 (69.2)	25 (73.5)	28 (66.7)	.52	28 (77.8)	24 (63.2)	.17	“She died in the operating room.…I still feel terrible about it, obviously…and I think of her around every Easter.”
I find myself repeatedly thinking about these types of events.	64 (82.1)	29 (85.3)	34 (81.0)	.62	34 (94.4)	27 (71.1)	.008	“I’m checking my phone all the time. And I’m thinking about what’s going on and calling the residents and they’re calling me and I’m trying to help my kids with homework and I’m thinking about my patient and I’m trying to sit and have dinner with them and hear about their day and I’m thinking about my patient.”
The mental weight of my experience is exhausting.	48 (61.5)	22 (64.7)	25 (59.5)	.64	26 (72.2)	21 (55.3)	.13	“The first and only time I’ve ever been sued was also a very psychologically scarring event, which no one tells you is incredibly isolating, because it has to be, and it lasts a long time.”
My experience with these occurrences can make it hard to sleep regularly.	20 (25.6)	13 (38.2)	7 (16.7)	.03	11 (30.6)	8 (21.1)	.35	“It’s one of those things where you wake up in the middle of night, you’re like, checklist. Could I have done this? Could I have done this? Could I have done this?”
The stress from these situations has made me feel queasy or nauseous.	20 (25.6)	13 (38.2)	6 (14.3)	.02	6 (16.7)	12 (31.6)	.14	NA
Thinking about these situations can make it difficult to have an appetite.	18 (23.1)	13 (38.2)	4 (9.5)	.003	7 (19.4)	11 (29.0)	.34	NA
Following my involvement, I experienced feelings of inadequacy regarding my patient care abilities.	65 (83.3)	32 (94.1)	32 (76.2)	.03	33 (91.7)	29 (76.3)	.07	“The immediate emotions were guilt, self-doubt…”
My experience makes me wonder if I am not really a good health care provider.	59 (75.6)	31 (91.2)	27 (64.3)	.006	28 (77.8)	28 (73.7)	.68	“Initially I was like, oh, I’m a terrible doctor.”
After my experience, I became afraid to attempt difficult or high-risk procedures.	51 (65.4)	25 (73.5)	25 (59.5)	.20	31 (86.1)	18 (47.4)	<.001	“You [can] then say, well, I’m just not gonna do these complicated cases. Some people do that. They’re just like…I’m not doing complicated tumor cases or trauma cases. And fair enough.”
My experience with these events has led to a desire to take a position outside of patient care.	21 (26.9)	11 (32.4)	10 (23.8)	.41	13 (36.1)	7 (18.4)	.09	“It was a little bit fortuitous.…I did more administrative things, I wasn’t as clinically active.”
Sometimes the stress from being involved with these situations makes me want to quit my job.	28 (35.9)	15 (44.1)	12 (28.6)	.16	14 (38.9)	13 (34.2)	.68	“I think that can really affect your confidence, your ability to keep going and progressing and wanting to do the field.…In all honesty, I would not have gone into this field if I had to do it again.”

Abbreviation: NA, not applicable.

^a^ Denominators differ for some statements because some participants did not answer all items.

### Disparities by Gender and Racial and Ethnic Background

When stratified by gender, questions describing the negative personal impact of adverse patient events consistently received more yes responses from female trainees compared with male trainees (Table 2). The greatest gender disparity was observed in response to the prompt, “My experience makes me wonder if I am not really a good healthcare provider” (31 of 34 female trainees [91.2%] vs 27 of 42 male trainees [64.3%], *P* = .006; effect size = 0.66; 95% CI, 0.19-1.12). Additionally, female trainees reported a significantly higher rate of physical symptoms compared with male trainees, including difficulty sleeping (13 of 34 female trainees [38.2%] vs 7 of 42 male trainees [16.7%], *P* = .03; effect size = 0.50; 95% CI, 0.04-0.96) and loss of appetite (13 of 34 female trainees [38.2%] vs 4 of 42 male trainees [9.5%], *P* = .003; effect size = 0.72; 95% CI, 0.25-1.19).

When stratified by race and ethnicity, trainees who identified as having a minority racial or ethnic background responded yes at higher rates than non-Hispanic White trainees on all but 2 questions (Table 2). The largest difference between groups was elicited in response to the statement, “After my experience, I became afraid to attempt difficult or high-risk procedures” (31 of 36 trainees with a minority race or ethnicity [86.1%] vs 18 of 38 non-Hispanic White trainees [47.4%], *P* < .001; effect size = 0.89; 95% CI, 0.40-1.36).

### Current and Desired Forms of Support

When asked about sources of support used in the wake of adverse events, trainees most commonly reported leaning on friends and family (69 of 78 trainees [8.5%]) and informal support from colleagues in the department (52 of 77 trainees [67.5%]). Only 4of 77 trainees (5.3%) had used formal institutional resources, such as the free counseling sessions, and 2 of 76 trainees (2.6%) had taken a “mental health day.”

When presented with potential options for support after an adverse event, trainees expressed the most interest in opportunities to discuss the case with an attending physician (76 of 78 respondents [97.4%]) or trusted senior trainee (73 of 78 respondents [94.8%]). Additionally, despite the low rate of current use, 23 of 78 respondents (29.5%) expressed a strong desire (“quite a bit” or “a lot”) for an employer-sponsored counseling program. The ability to take time off was strongly desired by 18 of 78 trainees (23.1%), and having a private space to recover was strongly desired by 29 of 78 trainees (37.2%).

### Part B: Faculty Interviews

Of 29 faculty invited to participate, 23 (79.3%) accepted and completed interviews. Participants had been in practice of a median (IQR) of 11.0 (7.5-20.0) years; 13 (56.5%) identified as male and 10 (43.5%) as female. We did not collect data on age, race, or ethnicity from participating faculty due to concerns about preserving anonymity in this small, single-institution study. No faculty interviewees declined to answer any of the interview questions; therefore, no interview data were missing. Several themes emerged within each of the 3 study domains and built on the results from the trainee survey.

### Personal Impact of Adverse Patient Events

All interviewees described at least 1 adverse event that was especially challenging from a personal standpoint. Many of the described events shared common characteristics that made them particularly salient: (1) the event was unexpected, (2) there was a lack of a clear explanation for what happened, and (3) the event led to significant harm to the patient. Mirroring the survey responses, most interviewees described feelings of embarrassment, guilt, shame, and self-doubt after these events. Many discussed being distracted by thoughts of the event both at work and at home, and some talked about how the complication took on an outsized role in their mind compared with other cases and activities. In contrast to the results from the trainee survey, few surgeons reported physical manifestations of their experiences with adverse events. However, adding another dimension beyond the trainee survey results, surgeons discussed how these events can have a lasting impact over time, both personally and professionally. Interviewees described how memories of the event tended to resurface indefinitely, and how they experienced a period of increased vigilance that only dissipated months to years later. Finally, several surgeons discussed how being involved in malpractice litigation perpetuated the emotional impact of the adverse event due to the associated isolation, delay in closure, and questioning of professionalism. Representative quotations from this domain are included in Table 2.

### Coping Strategies

Several themes emerged from interviewees’ discussion of their coping strategies, paralleling the responses at the trainee level (Table 3). Most interviewees had identified 1 or a few trusted colleagues, typically in the same subspecialty, to whom they could turn for perspective and reassurance. Early- and middle-career interviewees discussed the value of reviewing complications with senior mentors. Several interviewees emphasized that in order to access peer support, they had to actively seek it out. Some surgeons described feeling comfortable sharing details of cases with their peers, yet others stated that they were reluctant to reach out.

**Table 3.** Themes, Subthemes, and Representative Quotations From Domain 2 (Coping Strategies)

Subtheme	Representative quotations
**Affirmation from colleagues**	
Peer support	“She’s just my compatriot in this. She’s kind of the same stage in her career. She does exactly what I do, professionally. And so, we are able to talk to each other.”
	“And I got a lot of positive reinforcement from colleagues saying I’m not so sure I would have done anything different than what you did.”
Senior mentor	“My usual way of coping with it was to go to my senior partner, and I think of it as carrying my albatross into his office and laying it at his feet.…There was nothing that I would have done that he hasn’t done too. And just having that kind of person was extremely helpful to me, especially in my earlier years of practice, just being able to confess and share our shortcomings and be human. It’s kind of like a one-on-one M&M [morbidity and mortality review], and I thought it was very therapeutic.”
	“I went to talk to [Dr. X]. I said, listen, I need your help with this case. He says, you know what? You get back on the saddle. This happens. We’ll take care of it. Let’s fix it together. That kind of relationship is worth its weight in gold. And I think if a junior attending doesn’t have that kind of mentorship, it’s probably lonely as hell.”
Onus is on the surgeon to reach out	“There are definitely senior people that are available for you to talk to, but it is up to the individual surgeon to seek out these people.”
	“I have colleagues who probably have experienced the same thing, and I guess I could have easily talked to them, but I just didn’t. Because…you don’t want to burden other people with your stresses, especially when they may be experiencing the same stresses themselves.”
	“[My friend] published this piece about how distraught [she had] been over a patient event. I called and was like, ‘…You didn’t call me!’ And she was like, ‘I was so deep into the badness that I literally was afraid to even call you.’”
**Compartmentalization**	
Effective for some	“For me, personally, if I have something, I tend to kind of deal with it and not reach out.”
	“It’s just easier to function that way sometimes, when you sort of pack things away, and still be focused on something else.”
Insufficient for others	“If you don’t talk to other people about it, it just eats away at you.”
	“You can’t just put it inside. Some people can, but you’ve got to talk about it, because it’s just gonna be something you’re gonna face for your whole career.”
**Other strategies**	
Spousal support	“My husband is a great support, and we often talk about what’s going on at my job, but he’s not in medicine, so there’s only so much true empathizing.”
Reflection on positive outcomes	“I actually have saved some files of screenshots of very nice reviews that patients—as much as I hate that I’m being rated like a restaurant—I have saved some of them that are meaningful and kind. I will reread those to try and find a perspective.”
Hobbies	“I like to fly fish. So, it’s a getaway. I usually do it by myself, away from people and things. And so, it’s like a very sort of mindful, very relaxing sort of thing.”
Eschewing of mental health services	“I did not feel like I needed professional help or anything like that.”
	“I’ve never pursued any kind of psychological, psychiatric support, although people have told me to.”

Several additional coping mechanisms were discussed. A few interviewees described compartmentalization as their primary means of coping. Many surgeons spoke about receiving support from their spouses, but the degree of relief associated with spousal support varied depending on the spouse’s experience with the field of surgery. Some interviewees discussed turning to hobbies, such as exercise and fishing. A few mentioned reviewing notes of gratitude from other patients who had had a positive outcome. A few also mentioned using alcohol as a coping mechanism, although no surgeons discussed overuse or use of other substances. Notably, none of the interviewees discussed using professional mental health support services; in fact, several surgeons explicitly eschewed the idea of such services.

### Current and Suggested Institutional Support Systems

Echoing the small percentage of trainees who reported using formal institutional resources, most faculty were unfamiliar with—and skeptical of—such resources. Those who did name formal resources mentioned the Department of Psychiatry and an online wellness platform accessible by all health system employees. Several interviewees noted that a morbidity and mortality conference does not serve as a support mechanism for dealing with the personal impact of adverse events.

In describing the informal culture in their departments with regard to the personal impact of adverse events, surgeons were divided by specialty. Some described a sense of camaraderie and nonjudgment within their department; interestingly, most of these interviewees had trained at the study institution. Conversely, other surgeons described a culture of stigma, where faculty do not tend to discuss complications with each other for fear of reputational damage.

Most interviewees felt that more institutional support for faculty dealing with adverse patient events would be beneficial. Several interviewees spoke about the need for destigmatization. Additionally, many surgeons thought that having a designated point person for debriefing of adverse events would be helpful, but there was uncertainty about what type of individual would be best for that role. Suggestions included a senior faculty member with patient safety experience, an emeritus faculty member, and a trained therapist.

Similarly, most surgeons thought that there should be more explicit support for trainees regarding the issue of adverse patient events. Several interviewees discussed the importance of debriefing complications with trainees, but some noted that this process may need to be performed by a third party when the involved faculty member is unable to engage due to his or her personal involvement. Furthermore, surgeons observed that developing a process for coping with adverse events requires time and experience, and several suggested that training programs should take a more active role in setting expectations and helping trainees learn to deal with the personal impact. The themes and quotations from this domain are summarized in Table 4.

**Table 4.** Themes, Subthemes, and Representative Quotations From Domain 3 (Current and Suggested Institutional Support Systems)

Subtheme	Representative quotations
**Perceptions of current resources**	
Lack of formal resources	“Is there support within our department or division? I don’t know. That’s a great question. No one ever talks about it.”
	“There’s no formal mechanism for us to discuss it outside M&M….The obsession in M&M is, how could you have prevented it, rather than…how is the team handling that? So, yeah, I would say effectively zero from a formal standpoint.”
Skepticism	“So, there are resources that people can tap into. Whether they choose to do so or not is really an individual thing.”
	“I have never utilized it. I don’t even know if a resource like that exists. I’m not sure many would use it.”
**Stigma**	
Problematic for some	“People don’t talk to each other about these sorts of things. They just—whether it’s complications or even things like, when you’re young attending, the first time you get sued—people just don’t talk about it. It’s like a dirty little secret or something.”
	“My network would not be within my department. And perhaps some of that is because I would have concerns about potential retribution or—not retribution, but I don’t want it accounted for. Or, as much as people talk about things being confidential, I would have a fear it wasn’t.”
Less problematic for faculty who trained at same institution	“I think our division…is very supportive. I suspect that part of that is because I trained here, so I have some established relationships.”
	“I am fortunate to know and trust my colleagues because I had already been a member of the department for 9 years.…I could very easily see how that might be difficult if I entered a brand-new space without those established and trusted relationships.”
**Suggestions for faculty support**	
Destigmatization	“We need to be more open about discussing these things. And I think that openness will be the first step.…We have to develop a mindset that it’s okay to talk about these things.”
	“What you want is a culture of understanding.…If your name is up on the M&M board multiple weeks in a row, that you’re not gonna get the reputation as the attending that’s always causing problems.”
Point person for debriefing	“Having maybe 1 designated person who is familiar with safety events and talking to risk management.…Having a go-to person would probably be helpful.…It sort of normalizes and kind of destigmatizes the fact that everybody makes errors.”
	“I’ve heard of some departments having even a departmental therapist.…I’m not sure that exists. It sounds like it should. I feel like too often in our training within our field, we’re so caught up on the technical, on the medical side of things, that we forget to also look at the clinician themselves and say…if their mind isn’t straight, how do you expect them to function at the highest level?”
	“That might be a really good role for someone at the emeritus level.”
**Suggestions for trainee support**	
Event-specific outreach	“If you have a complication, I think it’s important that you talk to your resident afterwards.…Maybe that’s not done enough.”
	“I’ve had to remind myself to reach out and be like, ‘Hey, that was a tough case.’…But I don’t always feel like I’m the most qualified to help them cope because I’m already kind of struggling with it myself. I don’t have a great solution for what could be done. Maybe it’s alerting the program director like, hey, this happened, could you maybe just check in with them over the course of the week and see how they’re doing.”
	“I think that’s a very important role for the program directors.…Not mandatory, but they should at least be offered…a debriefing. Let’s just sit down and talk about this in a nonjudgmental way. I think that would be very important for the trainees.”
Formal instruction	“But I do think that having something more formal for trainees is more important because they have not yet had a chance to develop their own system for dealing with these issues.”
	“When you are a medical professional and you’re putting yourself in harm’s way emotionally, you need to be taught how to deal with that.”

Abbreviation: M&M, morbidity and mortality review.

## Discussion

In this mixed-methods study at an academic medical center, we found that most surgical trainees had been involved in at least 1 recent adverse event and subsequently experienced a negative personal impact. Despite low statistical power, female trainees and those from racial and ethnic minorities consistently reported negative consequences at a higher rate than male and non-Hispanic White trainees. Although only about half of trainees received support from colleagues after adverse events, nearly all who had been negatively impacted by an adverse patient event desired an opportunity to debrief with an attending or trusted senior resident. These findings were echoed in the themes that emerged from the faculty interviews. Surgeons universally described distress associated with adverse events and discussed how these cases often had a lasting personal impact. Surgeons differed in their perceptions of the institutional culture around adverse events by department; some described a culture of stigma, whereas others described a supportive and nonjudgmental culture. All agreed that departments and training programs could do more to help individuals manage the negative personal consequences of adverse events.

Our findings regarding the emotional and cognitive impact of adverse events on surgeons and trainees build on the existing literature. Two survey studies demonstrated that surgeons frequently experience negative emotions, such as anxiety (48%-66%) and anger (29%-32%), as well as guilt (60%) and embarrassment (42%), in the wake of complications.^30,31^ Similarly, 2 qualitative studies found that surgeons commonly experienced guilt, self-doubt, and rumination.^16,17^ One survey study of surgeons and trainees found that 36% met criteria for clinically concerning traumatic stress after a major complication.^32^

However, no prior studies have attempted to identify whether trainees or surgeons experience adverse events differently based on gender, race, or ethnicity. The differences we observed in surgical trainees’ reactions to adverse events by gender and racial or ethnic background is an important finding for the surgical workforce. Physicians who report higher levels of stress and anxiety are more likely to leave their practice or direct patient care altogether, and prior studies have revealed that rates of attrition from surgical training programs are significantly higher among female residents and those from underrepresented minorities.^13,33,34,35,36,37^ Thus, it is possible that the disproportionate burden of negative emotions and self-doubt experienced by female trainees and those from minority racial and ethnic backgrounds in the aftermath of adverse patient events represents a key contributor to the observed differences in attrition.

One of the major themes that emerged from the faculty interviews was the value of affirmation from colleagues, a concept that has been identified in previous work.^30,38,39^ However, the ability to access peers and mentors for support may differ by background. Because women and individuals of races and ethnicities other than non-Hispanic White have been historically underrepresented in surgical specialties, they may have fewer close social connections and trusted mentors with whom they identify and to whom they can turn for validation.^40^ Prior work in the medical student population has found that Black students enjoy less social support and less frequently identify with the stereotype of a surgeon.^41,42^ Similarly, female surgical trainees have been found to have higher rates of imposter syndrome than male trainees.^43,44^ Taken together, these findings suggest that formalizing mechanisms to provide peer support in the wake of adverse events may be particularly beneficial for surgeons and trainees from underrepresented groups.

Currently, our institution offers a web-based platform to support the mental health of all employees. Resources offered through the platform include podcasts, group mindfulness sessions, opportunities to schedule free appointments with resilience coaches and/or counselors from the Employee Assistance Program, and more. Although the platform does not track usage by role to protect anonymity, our data suggest that general, health system–wide support programs may be underused by surgeons.^9,45,46,47^ Thus, a dedicated approach to supporting surgeons may be warranted. One study of a surgeon-specific peer support program found that most respondents felt better after debriefing an adverse event with a trained colleague and would recommend the program to others.^47^ Although standing up a full peer support program requires significant time and effort, surgical departments could start by appointing a designated member to provide proactive outreach and support to faculty and trainees in the aftermath of serious adverse patient events. Given the culture of stigma and skepticism of formal support resources described by surgeons, any new institutional structure will require thoughtful implementation to ensure surgeon buy-in.^16,17,30^

Another important theme that emerged is that surgical training programs ought to explicitly help trainees develop strategies for coping with adverse events. This finding is consistent with 2 prior survey studies, which found that trainees felt unprepared to cope with the personal impact of complications and would welcome formal instruction.^48,49^ There are several potential ways in which surgical training programs could incorporate this component of professional development. For instance, setting expectations about the inevitability of adverse events early in training would likely promote destigmatization. Additionally, using educational conference time to discuss nontraditional topics, such as coping with adverse outcomes and dealing with medical malpractice issues, could help trainees proactively prepare for these events as well as expose them to faculty champions who could serve as resources in the future. With regard to formalizing support in the immediate aftermath of adverse patient events, programs could consider providing standard outreach to all trainees involved in in-hospital deaths and perhaps even to all trainees who submit complications that qualify as Clavien-Dindo grades IIIb or higher (ie, intervention under general anesthesia, life-threatening complication requiring intensive care management, or death) for morbidity and mortality review. As implied by our interview data, program directors themselves may have varying levels of comfort with this subject matter, and some training programs may have a more formal culture in which trainees are reluctant to display vulnerability in the presence of faculty with traditional leadership roles. Therefore, programs should individually assess their local culture using anonymous trainee input to identify 1 or more faculty members whose outreach would be most welcomed after adverse patient events. Finally, programs should regularly remind trainees about the available mechanisms for support and work toward greater concordance between the forms of support that trainees desire and those that are actually used.

We look forward to building on this work by partnering with surgical training programs at other institutions that do not yet have formal support structures for trainees. By serially readministering the trainee survey while implementing the interventions using a pragmatic stepped-wedge design, we hope to assess whether these efforts measurably mitigate the negative personal impact of adverse events.

### Limitations

This study has several limitations. First, its generalizability is limited because it reflects the experience of individuals at a single academic institution in the US. Second, use of the survey format for trainees may have introduced nonresponse bias and limited our ability to elicit nuance and creative suggestions. Third, our ability to detect statistically significant differences between subgroups was limited by the sample size; thus, future multi-institutional efforts are warranted. Fourth, in the faculty interviews, we did not explicitly ask about the role of gender and race in their experiences due to our desire to preserve anonymity.

## Conclusions

In this mixed-methods study of the personal impact of adverse patient events on surgeons and trainees, these cases conferred significant distress for the individuals involved, and trainees from underrepresented groups experienced greater negative consequences. To cope, both trainees and faculty rely on support from peer and senior colleagues. More formal support mechanisms at all levels may help decrease stigma and restore confidence. One surgeon perhaps said it best: “If their mind isn’t straight, how do you expect them to function at the highest level?”

## Notes

Supplement 1. **eAppendix 1.** Trainee Survey Instrument **eAppendix 2.** Faculty Interview Guide

Supplement 2.
Data Sharing Statement
