# Supplementary material for: Surgeon and Surgical Trainee Experiences After Adverse Patient Events
Source: JAMA Netw Open. 2024 Jun 3;7(6):e2414329. doi: 10.1001/jamanetworkopen.2024.14329 (PMC11148685; doi:10.1001/jamanetworkopen.2024.14329)
Supplement: Supplement 1. — eAppendix 1. Trainee Survey Instrument eAppendix 2. Faculty Interview Guide [file jamanetwopen-e2414329-s001.pdf]

# Supplementary Online Content

Ginzberg SP, Gasior JA, Passman JE, et al. Surgeon and surgical trainee experiences after patient adverse events: a mixed-methods study. *JAMA Netw Open*. 2024;7(6):e2414329. doi:10.1001/jamanetworkopen.2024.14329

**eAppendix 1.** Trainee Survey Instrument

**eAppendix 2.** Faculty Interview Guide

This supplementary material has been provided by the authors to give readers additional information about their work.

© 2024 Ginzberg SP et al. *JAMA Network Open.*

**eAppendix 1.** Trainee survey instrument, adapted from the Second Victim Experience and Support Tool originally created and validated by Burlison et al (2017).

1. Have you been involved in at least one adverse patient event (an error or unexpected negative outcome) in the last 12 months at Penn?
   - Yes
   - No

2. Approximately how many of these events have you experienced in the past 12 months?
   - 1
   - 2
   - 3
   - 4
   - 5+

| | Not at all | A little | Some-what | Quite a bit | A lot |
|---|---|---|---|---|---|
| 3. I have experienced embarrassment from these instances. | | | | | |
| 4. My involvement in these types of instances has made me fearful of future occurrences. | | | | | |
| 5. My experiences have made me feel miserable. | | | | | |
| 6. I feel deep remorse for my past involvements in these types of events. | | | | | |
| 7. I find myself repeatedly thinking about these types of events. | | | | | |
| 8. The mental weight of my experience is exhausting. | | | | | |
| 9. My experience with these occurrences can make it hard to sleep regularly. | | | | | |
| 10. The stress from these situations has made me feel queasy or nauseous. | | | | | |
| 11. Thinking about these situations can make it difficult to have an appetite. | | | | | |
| 12. I appreciate my coworkers' attempts to console me, but their efforts can come at the wrong time. | | | | | |

© 2024 Ginzberg SP et al. *JAMA Network Open.*

| | Not at all | A little | Some-what | Quite a bit | A lot |
|---|---|---|---|---|---|
| 13. Discussing what happened with my colleagues provides me with a sense of relief. | | | | | |
| 14. My colleagues can be indifferent to the impact these situations have had on me. | | | | | |
| 15. My colleagues help me feel that I am still a good healthcare provider despite any mistakes I have made. | | | | | |
| 16. My department understands that those involved may need help to process and resolve any effects they may have on care providers. | | | | | |
| 17. My department offers a variety of resources to help me get over the effects of involvement with these instances. | | | | | |
| 18. The concept of concern for the well-being of those involved in these situations is not strong in my department. | | | | | |
| 19. I look to close friends and family for emotional support after one of these situations happens. | | | | | |
| 20. Following my involvement, I experienced feelings of inadequacy regarding my patient care abilities. | | | | | |
| 21. My experience makes me wonder if I am not really a good healthcare provider. | | | | | |
| 22. After my experience, I became afraid to attempt difficult or high-risk procedures. | | | | | |
| 23. These situations do not make me question my professional abilities. | | | | | |
| 24. My experience with these events has led to a desire to take a position outside of patient care. | | | | | |
| 25. Sometimes the stress from being involved with these situations makes me want to quit my job. | | | | | |

26. My experience with an adverse patient event has resulted in me taking a mental health day.
    - Yes
    - No

© 2024 Ginzberg SP et al. *JAMA Network Open.*

27. Have you been asked to discuss an adverse patient event in a Root Cause Analysis (RCA) or Morbidity & Mortality (M&M) conference in the past 12 months?
    - RCA
    - M&M
    - Both

28. How did your involvement in the RCA or M&M affect your emotional state regarding the adverse patient event?
    - Very negatively
    - Somewhat negatively
    - No difference
    - Somewhat positively
    - Very positively

29. I have received support after one of these events from the following Penn resources(s):
    - Penn colleagues (informally)
    - Employee Assistance Program
    - COBALT
    - Other _______
    - None, I have never received support from anyone at Penn

30. Please indicate your level of interest in the following types of support that could be offered by your department for those who have been negatively affected by an adverse patient event:

| | Not at all | A little | Some-what | Quite a bit | A lot |
|---|---|---|---|---|---|
| The ability to immediately take time off from my rotation | | | | | |
| A private location that is available to recover and recompose | | | | | |
| A discussion with a respected senior trainee about the incident | | | | | |
| A discussion with my attending about the incident | | | | | |
| An employee assistance program that can provide free counseling to employees outside of work | | | | | |
| A non-Penn therapist who can help me process the event | | | | | |

31. What else would you like to share about this topic?

| |
|---|
| |

© 2024 Ginzberg SP et al. *JAMA Network Open.*

32. What is your role?
    - Resident, PGY 1-2
    - Resident, PGY 3-4
    - Resident, PGY5+
    - Fellow

33. What is your specialty?
    - General Surgery
    - Urology
    - Plastic Surgery
    - Vascular Surgery
    - Cardiac Surgery
    - Orthopedic Surgery
    - Otorhinolaryngology
    - Neurosurgery
    - Interventional Radiology
    - Internal Medicine
    - Psychiatry
    - Prefer not to answer

34. Gender identity:
    - Female
    - Male
    - Gender non-binary
    - Other
    - Prefer not to say

35. Ethnicity:
    - Hispanic or Latinx
    - Not Hispanic or Latinx
    - Prefer not to answer

36. Race (check all that apply):
    - Black or African-American
    - White
    - Asian or Pacific Islander
    - Other
    - Prefer not to answer

37. Relationship status
    - Married
    - In a committed relationship
    - Separated
    - Single
    - Prefer not to answer

© 2024 Ginzberg SP et al. *JAMA Network Open.*

**eAppendix 2.** Faculty interview guide.

1. Think of an adverse patient event that impacted you personally and/or professionally. Tell me a bit about the case.
    a. *If not addressed:* How long ago did this event occur?
2. How would you describe your thoughts and feelings after the event?
3. How did you cope with the event?
    a. *If not addressed:* Who did you talk to about the event, and how did those conversations affect you?
4. How did the event affect your home life and what you do outside of work?
5. How did this event affect your approach to future cases?
6. How has your reaction to the event evolved over time, and how do you see it now?
7. How does your experience with this particular case compare to your experiences with other adverse patient events?
8. How would you describe the level of support in your division and/or department when a surgeon experiences an adverse patient event that affects them personally?
9. What do you think your division and/or department should do to support faculty when they experience an adverse patient event?
    a. How about trainees?
10. What else would you like to share about your experience?

© 2024 Ginzberg SP et al. *JAMA Network Open.*
