# Supplementary material for: Surgeon and Surgical Trainee Experiences After Adverse Patient Events
Source: JAMA Netw Open. 2024 Jun 3;7(6):e2414329. doi: 10.1001/jamanetworkopen.2024.14329 (PMC11148685; doi:10.1001/jamanetworkopen.2024.14329)
Supplement: Supplement 2. — Data Sharing Statement [file jamanetwopen-e2414329-s002.pdf]

Data Sharing Statement

Ginzberg. Surgeon and Surgical Trainee Experiences After Patient Adverse Events. *JAMA Netw Open*. Published June 03, 2024. doi:10.1001/jamanetworkopen.2024.14329

Data
Data available: Yes
Data types: Deidentified participant data
How to access data: By request only: sara.ginzberg@pennmedicine.upenn.edu
When available: With publication

Supporting Documents
Document types: None

Additional Information
Who can access the data: Researchers whose proposed use of the data has been approved
Types of analyses: Related work
Mechanisms of data availability: After approval of a proposal and with a signed data access agreement
